# Commentary: Acute Tension-Type Headaches Are Associated with Impaired Cognitive Function and More Negative Mood

**DOI:** 10.3389/fneur.2016.00103

**Published:** 2016-06-30

**Authors:** Marcelo M. Valença

**Affiliations:** ^1^Neurosurgery and Neurology Unit, Federal University of Pernambuco, Recife, Pernambuco, Brazil

**Keywords:** headache disorders, primary, moon, tension-type headache, severity, performance

I found Smith’s article (1) extremely interesting. The author reported that an acute attack of tension-type headache (TTH) may be “associated with an increase in negative affect, poorer performance on working memory and semantic memory tasks, slower psychomotor performance, and increased distraction from irrelevant stimuli” (1).

Although there are a number of articles dealing with cognition/behavior in headache patients (2–4), there is still a consensus that the two most frequent primary headaches, migraine and TTH (5), do not significantly affect cognitive functions to a degree that daily activities are seriously affected, particularly when one considers the interictal periods.

It is well known that migraine attacks are incapacitating, whereas the TTH attacks are mild or moderate in intensity, not affecting the activities of everyday life. In fact, patients with TTH may suffer severe headache attacks, resulting in impairment of routine activities. In a study carried out in our university (6), involving 121 female nurses, 536 headache attacks were registered, 117 with features of TTH and 10 of probably TTH, 3 of these 127 attacks (2.4%) were considered severe.

Considering that headache attacks are relatively frequent, and the fact that they may impair cognitive function, the physician should consider prophylactic measures to avoid triggering attacks during the performance of important tasks, in particular, those that demand a high level of attention and precision.

The interesting results obtained by Smith (1) need, however, to be confirmed by other works with a larger number of subjects. In addition, the current different treatments that may be used as preventive forms to control the TTH attacks are usually not corroborated by evidence-based medicine.

When we are dealing with children suffering from TTH, which are regarded as a prevalent and debilitating condition for both child and his or her family, the parents are often reluctant to accept a prescription with a pharmacological agent (7). They tend to prefer a non-pharmacological treatment as their first choice (7). One of the approaches to be tried in children is to stimulate a healthy lifestyle in order to avoid factors that could trigger headaches at both home and school (e.g., a good quality of sleep, adequate water and food ingestion, physical activity, and learning how to cope with psychosocial stressors) (7).

There are various other non-pharmacological strategies that may be adopted to reduce the possibility of a headache attack (Figure 1). Acupuncture, manipulation, joint range of motion, massage, cold packs, home exercise programs, advice on posture, muscle stretching techniques, retraining, and razor are some alternatives that might be of use (8). A recent update of a Cochrane review concluded that acupuncture is effective for treating frequent episodic or chronic TTHs (9).

**Figure 1:**
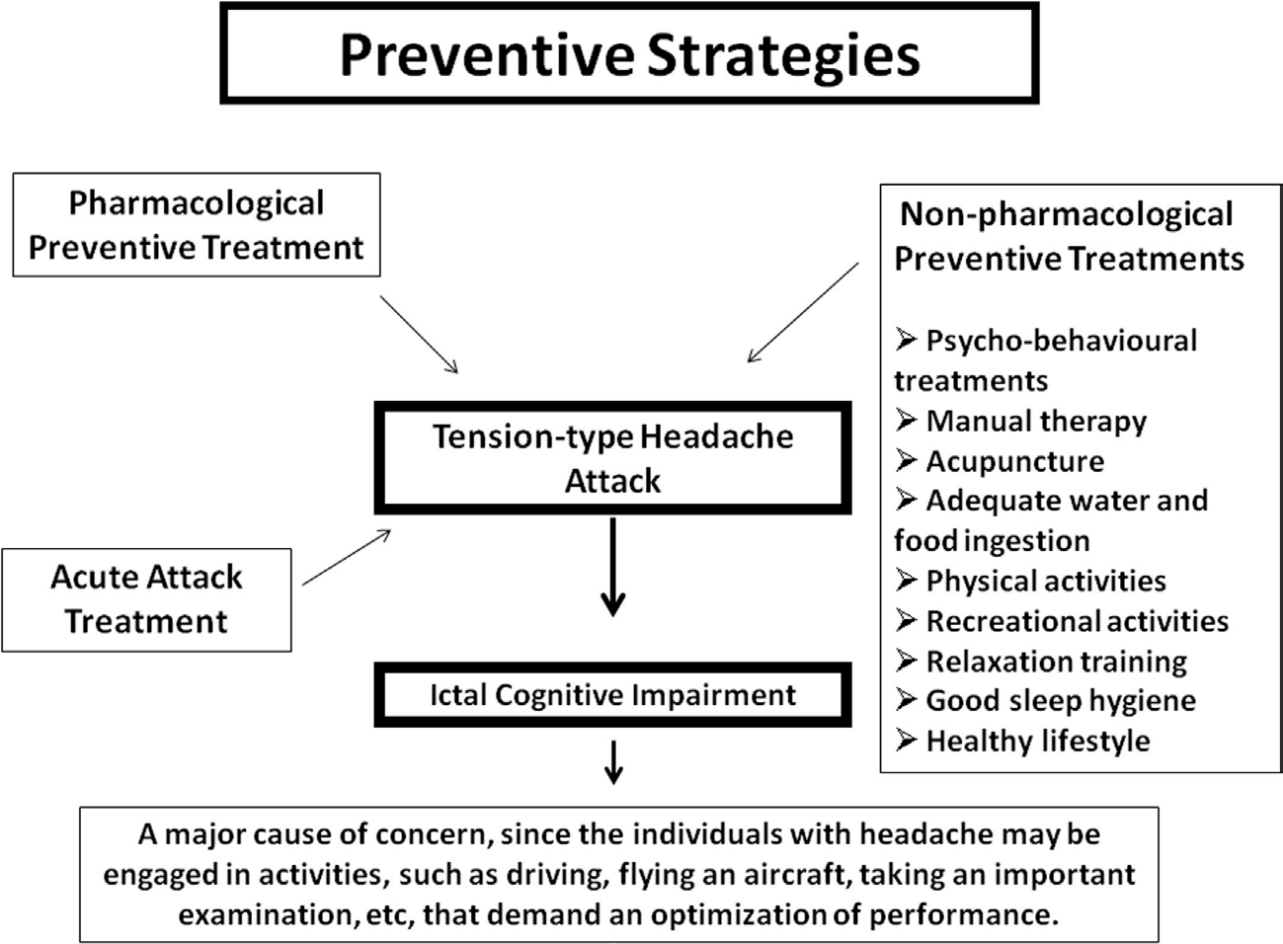
**Pharmacological and non-pharmacological strategies that may be adopted to reduce the possibility of a tension-type headache attack, which may impair cognitive function, representing a major cause of concern, when the individual is engaged in activities that demand an optimization of performance**.

Since the major concern here is the cognitive impairment during headache attacks, the use of preventive drugs to avoid such crises must bear in mind a possible additional cognitive deficit triggered by the choice of medication. The most widely used drug for treating TTH is amitriptyline, which, although it may attenuate a negative mood, can in fact worsen the cognitive symptomatology (10). There are a number of other pharmacological agents that have been employed as prophylactic treatment, such as antiepileptic drugs (sodium valproate, topiramate, and gabapentin), benzodiazepines, botulinum toxin, noradrenergic and specific serotonergic antidepressants (mirtazapine), serotonin re-uptake inhibitor antidepressants, and other tricyclic antidepressants (11); however, memory impairment has been reported with some of them.

A recent review (12) concluded that, in epileptic patients, some antiepileptic drugs, namely phenobarbital, phenytoin, topiramate, and zonisamide, can impair cognitive function. On the other hand, sodium valproate, carbamazepine, gabapentin, and oxcarbazepine do not appear to affect cognition. In addition, phenobarbital, valproate, gabapentin, topiramate, levetiracetam, and zonisamide may produce adverse behavioral side effects, although carbamazepine was considered neutral by the authors (12).

Nevertheless, venlafaxine and SSRIs were no more effective than amitriptyline or placebo in reducing the attack frequency in chronic TTH (13).

Recent evidence suggests that it is indeed possible to protect cognitive functioning by pharmacoterapy, despite the fact that its effectiveness has not been demonstrated to date in children or adolescents. The newly introduced drugs, levetiracetam and lamotrigine, seem to induce positive cognitive effects, with an additional positive behavioral effect being observed with the latter.

Topiramate is another drug widely used to prevent migraine and has also been used to treat chronic TTH, but it is well established that it can cause a major deterioration in cognition (particularly in relation to verbal fluency, memory spans, and working memory) (14). Since levetiracetam may improve cognitive abilities, it was tried to overcome the cognitive impairment induced by topiramate using it in combination with levetiracetam. Unfortunately, they were unsuccessful, using an experimental model with rats (15). In this context, a small series of patients with topiramate-related cognitive and language dysfunction improved with donepezil (16).

The ethical, legal, and public policy implications of the use of drugs, such as donepezil, rivastigmine, galantamine, caffeine, nicotine, amphetamines, modafinil, and memantine to enhance cognition in cognitively healthy individuals is reviewed by Mehlman (17). In this concern, memantine (18) and dextroamphetamine (19) were used for prophylaxis of chronic TTH.

Over-the-counter medications are largely used in the acute therapy for TTH (20). A recent review reported that the number needed to treat values for being pain-free at 2 h compared with placebo were 8.7, 8.9, and 9.8, respectively, for paracetamol 1,000 mg, ibuprofen 400 mg, and ketoprofen 25 mg (21).

Another possible form to treat acute TTH in adults and children above 6 years is the local topical use of 10% peppermint oil in ethanol, whose beneficial effects are comparable to that of paracetamol or acetylsalicylic acid (22).

In conclusion, in the future, new drugs are likely to be introduced or the agents in the current therapeutic arsenal are to be used in combination in order to overcome possible adverse cognitive and/or behavioral side effects that might occur during preventive therapy in patients with incapacitating headaches. Headache attacks can indeed impair cognitive function, representing a major cause of concern, since these individuals may be engaged in activities, such as driving, flying an aircraft, taking an important examination, etc., which demand an optimization of performance. Thus, the physician should consider prophylactic measures to avoid triggering attacks during the performance of such tasks.

## Author Contributions

The author confirms being the sole contributor of this work and approved it for publication.

## Conflict of Interest Statement

The author declares that the research was conducted in the absence of any commercial or financial relationships that could be construed as a potential conflict of interest.
